# Supplementary material for: Field control of quasiparticle decay in a quantum antiferromagnet
Source: Nat Commun. 2024 Jan 11;15:125. doi: 10.1038/s41467-023-44435-0 (PMC10784460; doi:10.1038/s41467-023-44435-0)
Supplement: Supplementary file 1 — Supplementary Information [file 41467_2023_44435_MOESM1_ESM.pdf]

Supplementary Information for Field Control of Quasiparticle Decay in a Quantum Antiferromagnet

Shunsuke Hasegawa, Hodaka Kikuchi, Shinichiro Asai, and Zijun Wei
Institute for Solid State Physics, The University of Tokyo, Chiba 277-8581, Japan

Barry Winn and Gabriele Sala
Neutron Scattering Division, Oak Ridge National Laboratory,
Oak Ridge, Tennessee 37831, USA

Shinichi itoh
Institute of Materials Structure Science,
High Energy Accelerator Research Organization, Ibaraki 305-0801, Japan

Takatsugu Masuda
Institute for Solid State Physics, The University of Tokyo, Chiba 277-8581, Japan
Institute of Materials Structure Science,
High Energy Accelerator Research Organization, Ibaraki 305-0801, Japan and
Trans-scale Quantum Science Institute,
The University of Tokyo, Tokyo 113-0033, Japan

(Dated: November 25, 2023)

I. EXPERIMENTAL DATA

A. Integration ranges for inelastic neutron scattering spectra

The integration ranges for figures of inelastic neutron scattering spectra in this article are summarized in Table S1. For a spectrum sliced along an momentum-energy ($\mathbf{q} - \hbar\omega$) plane (false color map), the ranges perpendicular to $\mathbf{q}$, two directions from $2\mathbf{a}^* - \mathbf{b}^*$, $\mathbf{b}^*$, and $\mathbf{c}^*$, are displayed in reciprocal lattice unit (r.l.u.). For a constant $\mathbf{q}$ cut, the ranges at the $\mathbf{q}$ are shown.

TABLE S1. Integration ranges for inelastic neutron scattering spectra. The definitions of the high symmetry positions in the momentum space is shown in Fig. 1**b** and 1**c** in the matin article.

Figure	Spectrometer	Type of spectrum	$\mathbf{q}$ or q	$2\mathbf{a}^* - \mathbf{b}^*$	$\mathbf{b}^*$	$\mathbf{c}^*$
Fig. 1a	HRC	Sliced spectrum	K_1 - Γ	-	0.1	0.05
Fig. 1a	HRC	Sliced spectrum	Γ - M_1	0.05	-	0.05
Fig. 1a	HRC	Sliced spectrum	M_1 - K_2	-	0.05	0.05
Fig. 1a	HRC	Sliced spectrum	K_2 - K_3	0.05	0.05	-
Fig. 2a-2f	HYSPEC	Sliced spectrum	M_3 - M_4	-	0.04	0.2
Fig. 3a	HYSPEC	Constant $\mathbf{q}$ cut	(4/3, −2/3, 0)	0.04	0.04	0.2
Fig. 3b	HYSPEC	Constant $\mathbf{q}$ cut	(1, 0, 0)	0.04	0.04	0.2
Fig. S1(a)	HYSPEC	Constant $\mathbf{q}$ cut	(3/2, 0, 0)	0.04	0.04	0.2

B. Magnetic field dependence of constant q cut

Magnetic field dependence of the constant $\mathbf{q}$ cuts, the peak energies, and full width half maxima ($\mathrm{FWHM_L}$) of the peaks at M point are shown in Fig. S1(a)-S1(c). The peak energies are reproduced using linear extended spin wave theory (LESW), as were those at K point in Fig. 3c in the main article.

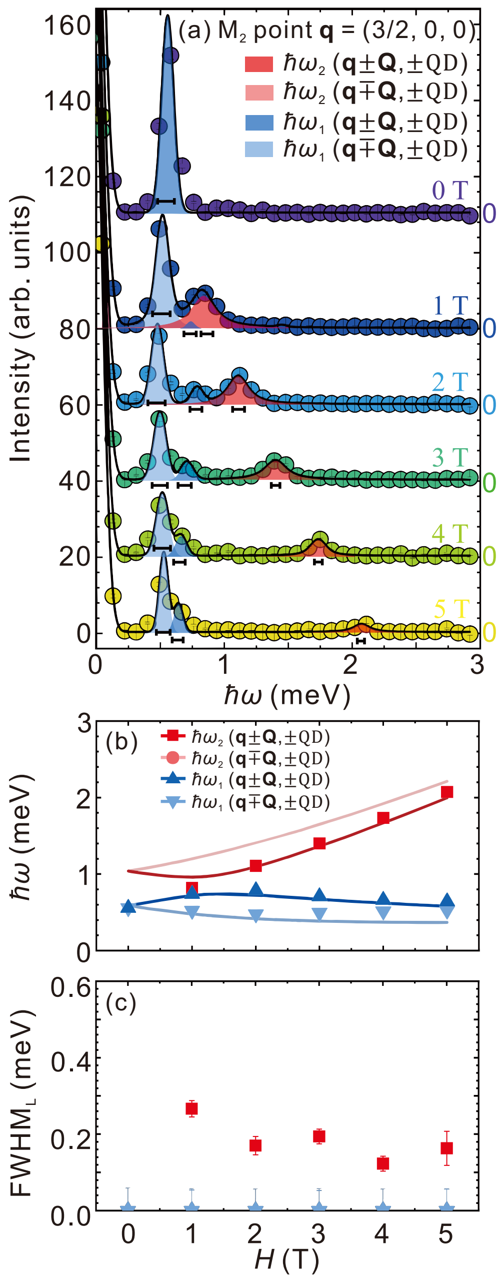

Fig. S1 (a) Magnetic field dependences of constant **q** cuts at M (3/2, 0, 0). The peaks indicated by deep and light blue are $\hbar\omega_1$ modes, and those by deep and light red are $\hbar\omega_2$ modes. The horizontal bars represent the instrumental resolution. (b) Magnetic field dependence of peak energies. Red and blue solid curves are calculations of $\hbar\omega_1$ and $\hbar\omega_2$ using linear extended spin wave theory. See Supplementary Information II B and Fig. S3 for the details of the modes in the legends. (c) Magnetic field dependence of full width half maximum ($\mathrm{FWHM_L}$). $\mathrm{FWHM_L} = 0$ means that the simple Gaussian function was employed for the fittings. Error bars for $\mathrm{FWHM_L} = 0$ represent instrumental resolution.

II. ANALYSIS

A. Inelastic neutron scattering cross-section in $RbFeCl_3$

We employ the effective spin $S = 1$ Hamiltonian:

$$\mathcal{H} = J_c \sum_{\langle i,j \rangle}^{\text{chain}} \mathbf{S}_i \cdot \mathbf{S}_j + J_{c2} \sum_{\langle i,j \rangle}^{\text{chain}} \mathbf{S}_i \cdot \mathbf{S}_j + J_{ab} \sum_{\langle i,j \rangle}^{\text{plane}} \mathbf{S}_i \cdot \mathbf{S}_j + \sum_i [D(S_i^z)^2 - g_c \mu_\text{B} H S_i^z], \quad \text{(S1)}$$

where J_c and J_{c2} are the nearest and next-nearest neighbor intrachain interactions, respectively; J_{ab} is the nearest neighbor interaction in the ab-plane, constructing triangular lattice; $D(>0)$ term is an easy-plane single-ion anisotropy, and the last term is the Zeeman term; the g value along the c direction is $g_c = 2.54$[1]. The exchange constants are indicated in Fig. S2.

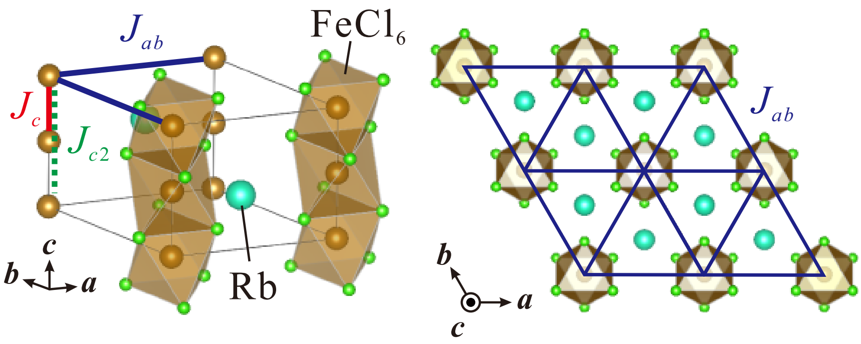

Fig. S2 The crystal structure and exchange constants in $RbFeCl_3$.

The standard spin wave theory in linear approximation fails to reproduce the inelastic neutron scattering (INS) spectrum even at 0 T[2]. Hence, we used LESW, where the three eigenstates of the mean-field Hamiltonian were employed as the basis functions: ground state, the first excited state which is excited by the transverse component of the spin operator, T state, and the second excited state which is excited only by the longitudinal component, L state at 0 T. In contrast to the standard spin wave theory, employing the eigenstate (which has longitudinal fluctuation) captures the quantum effect of strong easy-plane-type single-ion anisotropy and the hybridization between transverse and longitudinal fluctuation from a non-collinear magnetic structure[3–7]. In the present study we used the

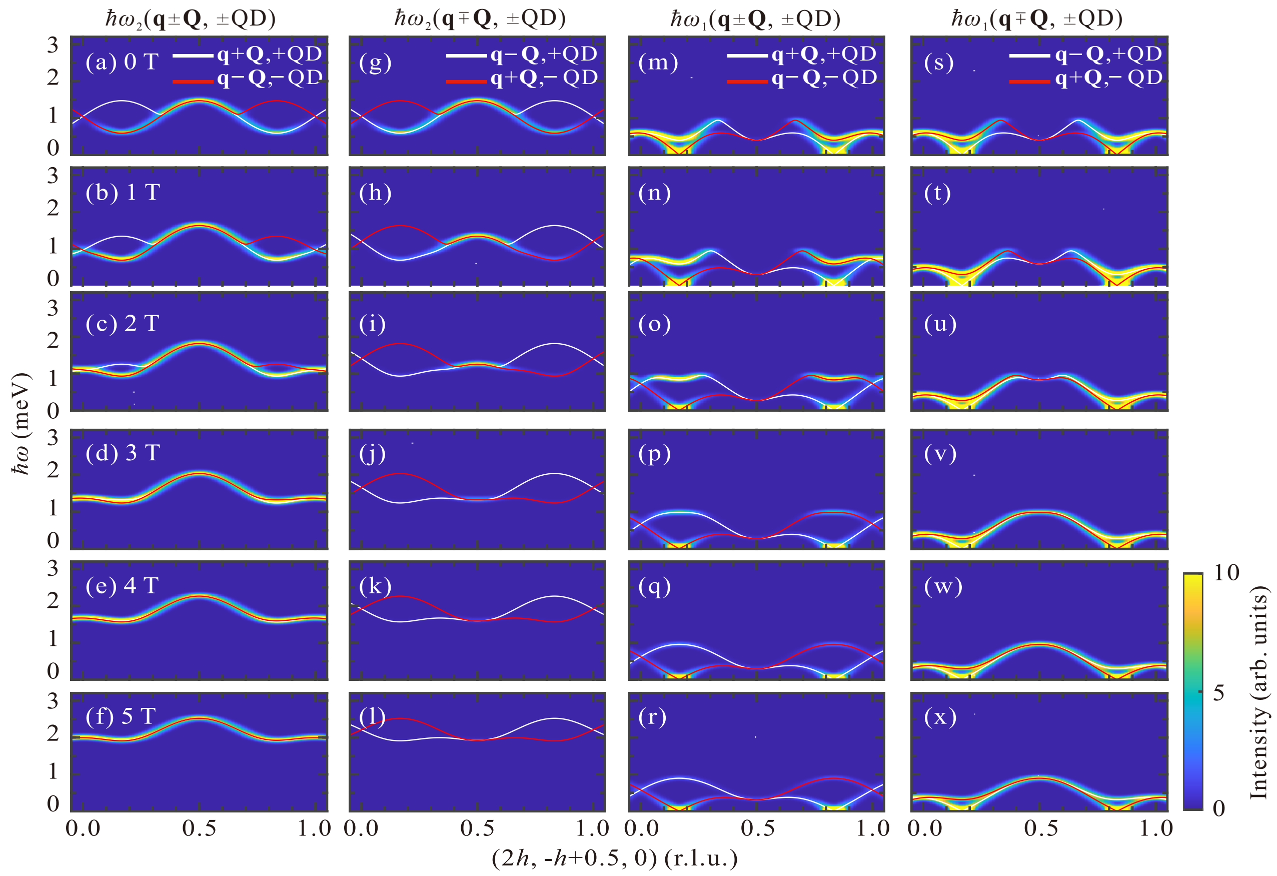

Fig. S3 Magnetic field dependences of inelastic neutron scattering cross-section for the pair of mode. White and red curves represent dispersion relations for +QD and −QD of each pair, respectively, where QD is the magnetic domain represented by $\mathbf{Q}$.

recipe described in Ref. 3 for the calculation. At 0 T, the low energy mode $\hbar\omega_1(\mathbf{q})$, high-energy mode $\hbar\omega_2(\mathbf{q})$, and their relatives $\hbar\omega_i(\mathbf{q}+\mathbf{Q})$ and $\hbar\omega_i(\mathbf{q}-\mathbf{Q})$ (i =1, 2) were derived (six modes in total). Here $\mathbf{Q}$ is the propagation vector, $\mathbf{Q} = (1/3, 1/3, 0)$. Fitting the dispersion relation to the data at 0 T with the parameters of J_c, J_{c2}, J_{ab}, and D is depicted by the curves in Fig. 1(a) in the main article. Successful fit using the calculation means that the use of L state as one of the basis functions in LESW was good and a substantial amount of longitudinal fluctuation was present in the observed spectra of $RbFeCl_3$. The parameters obtained were $J_c = -0.628(5)$ meV, $J_{c2} = 0.104(5)$ meV, $J_{ab} = 0.048(1)$ meV, and $D = 2.32(1)$ meV, which agrees with the previous study[8].

The excitations in all fields appeared to have the inversion symmetry with respect to the Γ point as shown in Figs. 2(a)-2(f) in the main article. This is explained by the superposition

of excitations from two magnetic domains defined by the propagation vectors $\pm\mathbf{Q}$. Magnetic domains represented by $+\mathbf{Q}$ will hereafter be referred to as +QD, and $-\mathbf{Q}$ as −QD. The application of a magnetic field perpendicular to the spin plane of the 120° structure induces a non-reciprocal magnon $\varepsilon(\mathbf{q}, +\mathrm{QD}) \neq \varepsilon(-\mathbf{q}, +\mathrm{QD})$ in each domain[3,9]. It was also found that the excitations from the two domains had the relation of $\varepsilon(\mathbf{q}, +\mathrm{QD}) = \varepsilon(-\mathbf{q}, -\mathrm{QD})$, and its superposition $\varepsilon_s(\mathbf{q}) = \varepsilon(\mathbf{q}, +\mathrm{QD}) + \varepsilon(\mathbf{q}, -\mathrm{QD})$ was reciprocal. The excitations from the two domains were observed to have equivalent scattered intensity, suggesting equivalent population for each domain. This result is similar to that reported in $Ba_3CoSb_2O_9$[10].

The degeneracy of modes from the two magnetic domains +QD and −QD at the zero field was lifted by applying the magnetic field. Twelve modes are obtained: $\hbar\omega_i(\mathbf{q}, \pm\mathrm{QD})$, $\hbar\omega_i(\mathbf{q}+\mathbf{Q}, \pm\mathrm{QD})$ and $\hbar\omega_i(\mathbf{q}-\mathbf{Q}, \pm\mathrm{QD})$ (i = 1, 2). Among these modes, the combination of $\hbar\omega_i(\mathbf{q}+\mathbf{Q}, +\mathrm{QD})$ and $\hbar\omega_i(\mathbf{q}-\mathbf{Q}, -\mathrm{QD})$, and $\hbar\omega_i(\mathbf{q}-\mathbf{Q}, +\mathrm{QD})$ and $\hbar\omega_i(\mathbf{q}+\mathbf{Q}, -\mathrm{QD})$ were related by inversion symmetry. The pairs of the combinations as modeled by LESW are exhibited in Figs. S3(a)-S3(x), with both dispersion and calculated cross sections. Owing to the strong single-ion anisotropy of the easy-plane type, the INS cross-section for $\hbar\omega_i(\mathbf{q}, \pm\mathrm{QD})$ (i = 1,2) was negligible, and they are not shown. The cross-sections of the pairs for $\hbar\omega_2$ in Figs. S3(b)-S3(f) and Figs. S3(h)-S3(l) were significant for only one of two branches in the high energy region at a given $\mathbf{q}$. Conversely, those for $\hbar\omega_1$ in Figs. S3(n)-S3(r) and S3(t)-S3(x) predicted scattered intensity for gapped and gapless branches in the low energy region. In total we have the four pairs of the gapped modes, $\hbar\omega_2(\mathbf{q}\pm\mathbf{Q}, \pm\mathrm{QD})$, $\hbar\omega_2(\mathbf{q}\mp\mathbf{Q}, \pm\mathrm{QD})$, $\hbar\omega_1(\mathbf{q}\pm\mathbf{Q}, \pm\mathrm{QD})$, and $\hbar\omega_1(\mathbf{q}\mp\mathbf{Q}, \pm\mathrm{QD})$, and two pairs of the gapless modes, $\hbar\omega_1(\mathbf{q}\pm\mathbf{Q}, \pm\mathrm{QD})$ and $\hbar\omega_1(\mathbf{q}\mp\mathbf{Q}, \pm\mathrm{QD})$. Sums of all 12 modes are presented in Figs. 2(g)-2(l) in the main article, which reasonably reproduced the observed spectra in Figs. 2(a)-2(f).

Extended spin wave theory treats longitudinal fluctuations within the linear approximation, leading to the successful semi-quantitative analysis on $RbFeCl_3$. However, the anharmonic term is not fully considered via LESW, leading to an underestimate of J_{eff} and an overestimate of D, where $J_{\mathrm{eff}} = 2(-2J_{c1} + 2J_{c2} + 3J_{ab})$. The observed ordered moment from the neutron diffraction experiment was reported to be 1.9 μ_{B} at 1.45 K[11], whereas the estimate from the mean-field solution in LESW was 0.94 μ_{B} using $g_{\mathrm{ab}} = 3.84$[12]. The underestimate of the magnetic moment leads to the evaluation of the ground state of the system as being closer to the quantum critical point (QCP) than reality. This corresponds to the

underestimate of J_{eff}/D[13]. The underestimate of J was reported in the standard linear spin wave theory as well[14]. The saturation field of the bulk magnetization in the field applied along the c-axis was 14 T[15], whereas the field estimated from LESW is 20 T. Because the saturation field reflects the magnitude of the easy-plane type anisotropy D, the overestimate of the field results in the overestimate of D. The true ground state of $RbFeCl_3$ is further from the QCP than estimated by the spin parameters from LESW; hence, the longitudinal component of the spin fluctuation would be overestimated. Please note that in the field control of magnon decay, the interaction between one-magnon and two-magnon continuum plays an important role, and the absolute values of the exchange constants are irrelevant. The underestimate of J_{eff} does not affect the discussion in the main article.

B. Cross section for longitudinal fluctuation

To illustrate the amount of longitudinal spin fluctuation, I_{XX}/I calculated using LESW, where I_{XX} is the cross section from the longitudinal spin correlation[3] and I is the total cross section, is shown for $\hbar\omega_1(\mathbf{q}{\pm}\mathbf{Q}, \pm\text{QD})$ and $\hbar\omega_1(\mathbf{q}{\mp}\mathbf{Q}, \pm\text{QD})$ in Fig. S4 and $\hbar\omega_2(\mathbf{q}{\pm}\mathbf{Q}, \pm\text{QD})$ and $\hbar\omega_2(\mathbf{q} \mp \mathbf{Q}, \pm\text{QD})$ in Fig. S5. The regions where the modes were not experimentally observed are hatched by gray. It was found that the $\hbar\omega_1$ modes were dominated by the transverse fluctuation, whereas $\hbar\omega_2$ modes had significant longitudinal fluctuation.

C. Two-magnon density of state

We calculated 2M-DoS, $\tilde{D}(\mathbf{q},\omega)$, by the sum of the 2M-DoS calculated in the local coordinate for each QD, $D(\mathbf{q},\omega,\pm\text{QD})$:

$$\begin{aligned}
\tilde{D}(\mathbf{q},\omega) &= \frac{1}{4}(D(\mathbf{q}-\mathbf{Q},\omega,+\text{QD}) + D(\mathbf{q}+\mathbf{Q},\omega,+\text{QD}) + D(\mathbf{q}-\mathbf{Q},\omega,-\text{QD}) \\
&+ D(\mathbf{q}+\mathbf{Q},\omega,-\text{QD})) \qquad \text{(S2)} \\
D(\mathbf{q},\omega,\pm\text{QD}) &= \frac{1}{N}\sum_{\mathbf{q}_1,\mathbf{q}_2,i,j}(\delta(\hbar\omega - \hbar\omega_i(\mathbf{q}_1,\pm\text{QD}) - \hbar\omega_j(\mathbf{q}_2,\pm\text{QD})) \\
&\times\ \delta(\mathbf{q}-(\mathbf{q}_1+\mathbf{q}_2)+\tau)), \qquad \text{(S3)}
\end{aligned}$$

where τ is a reciprocal vector and N is the number of spins. 2M-DoS is shown in Figs. 2(m)-2(r) in the main article.

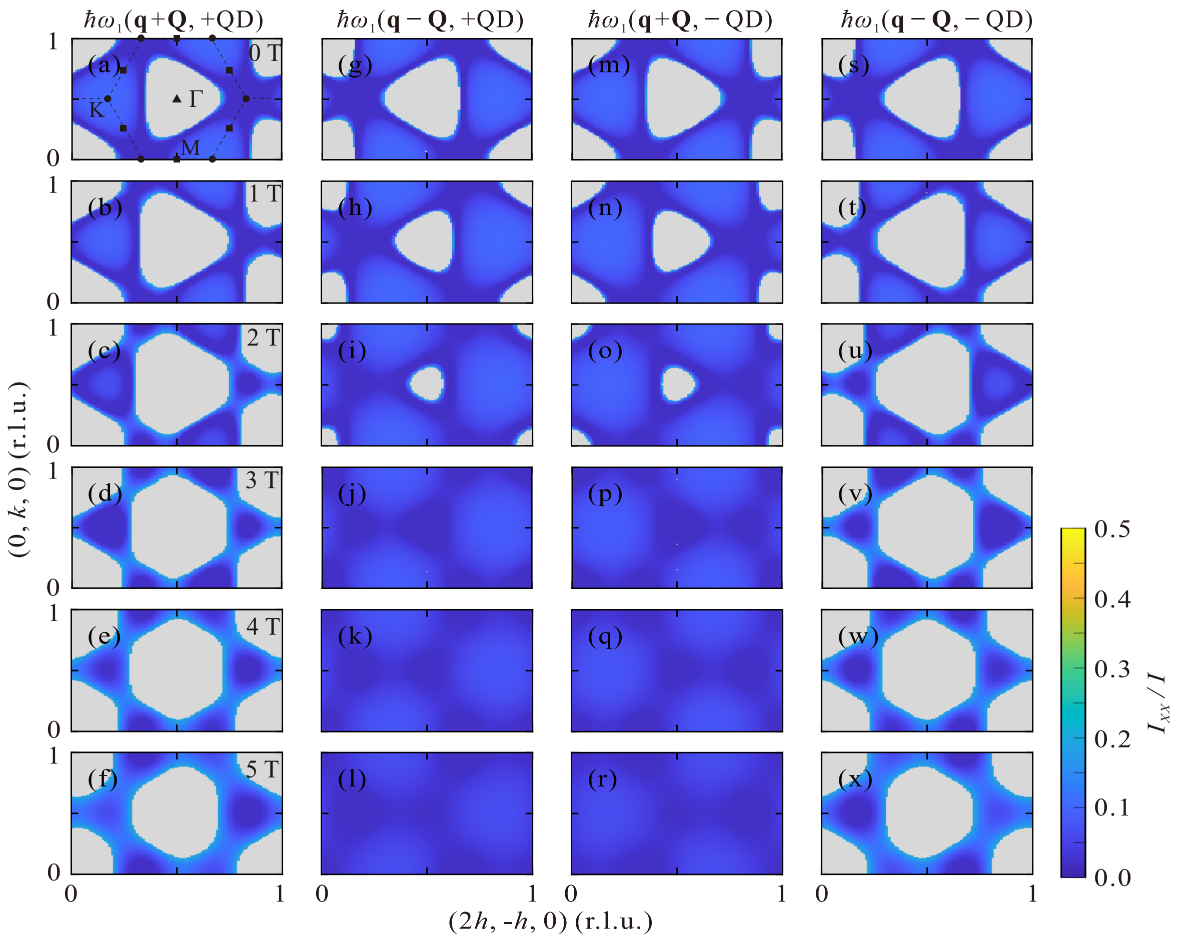

Fig. S4 Magnetic field dependences of I_{XX}/I for $\hbar\omega_1$ modes, where I_{XX} is the cross section from the longitudinal spin correlation and I is the total cross section. Dashed line represents Brillouin zone edge. Circles, squares, and triangles represent high symmetry point of K, M, Γ points, respectively. **q** region where modes were not experimentally observed are hatched by gray.

One-dimensional cuts of 2M-DoS in the magnetic fields at the Γ point are represented by blue lines in Figs. S6(a)-S6(f). At 0 T, the maximum of 2M-DoS was estimated at 1.8 meV. With the increase in magnetic field, the maximum energy increased, the peak sharpened, and the peak top increased. The onset of an additional peak appeared at 1.4 meV at 2 T. The additional peak energy decreased and the peak became sharper with the field.

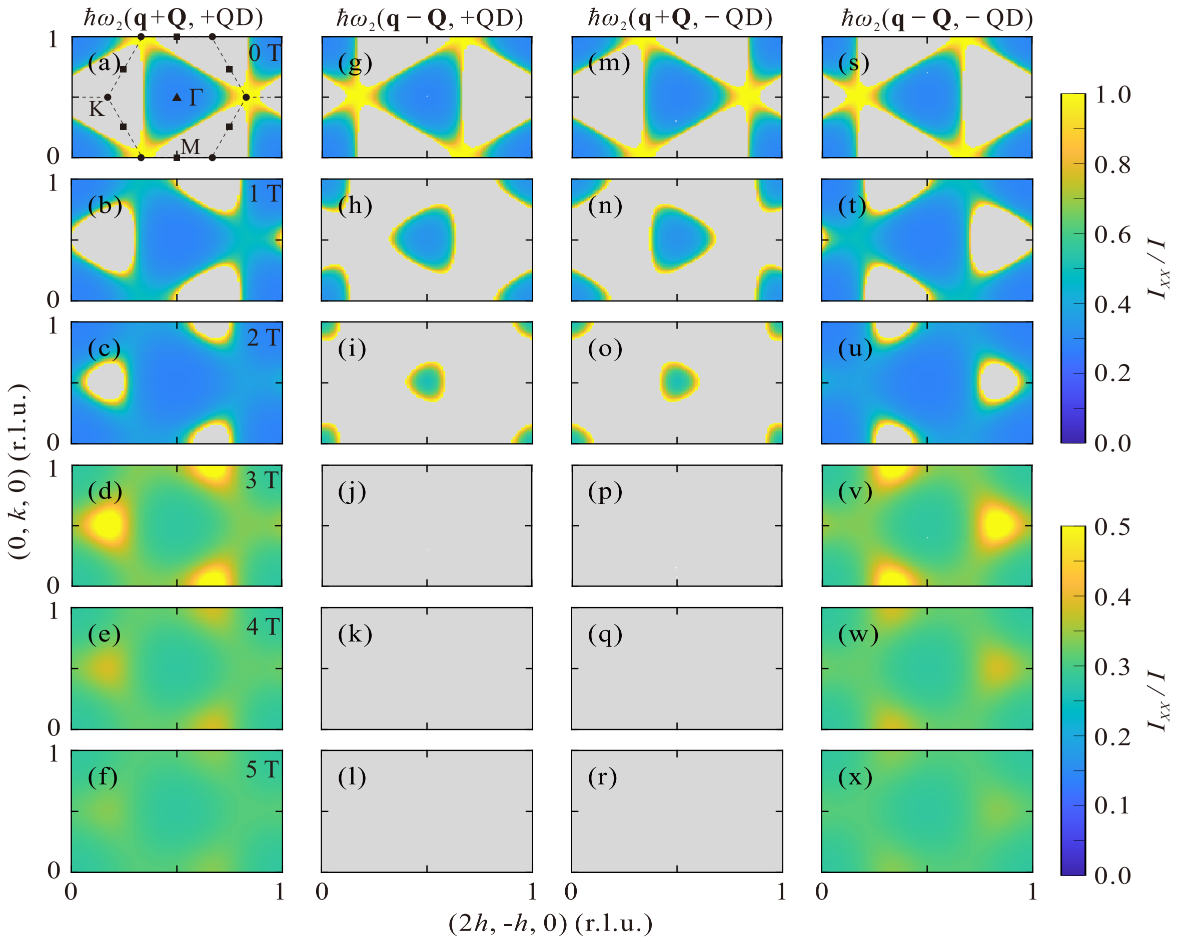

Fig. S5 Magnetic field dependences of I_{XX}/I for $\hbar\omega_2$ modes, where I_{XX} is the cross section from the longitudinal spin correlation and I is the total cross section. Dashed line represents Brillouin zone edge. Circles, squares, and triangles represent high symmetry point of K, M, Γ points, respectively. **q** region where the modes were not experimentally observed are hatched by gray.

III. INSTRUMENTAL RESOLUTION

Instrumental resolution has been modelled employing a Monte Carlo neutron ray-tracing software, MCViNE[16,17]. Three separate Monte Carlo simulations have been performed to estimate the contributions to the resolution given by the instrument optics, the sample, and the detectors. Specifically, we first simulate the neutron interactions with all the optics components present along the beam path such as guides, choppers, slits, and the monochromator, saving the output into a Monte Carlo particle list file 15 cm before the sample position. We

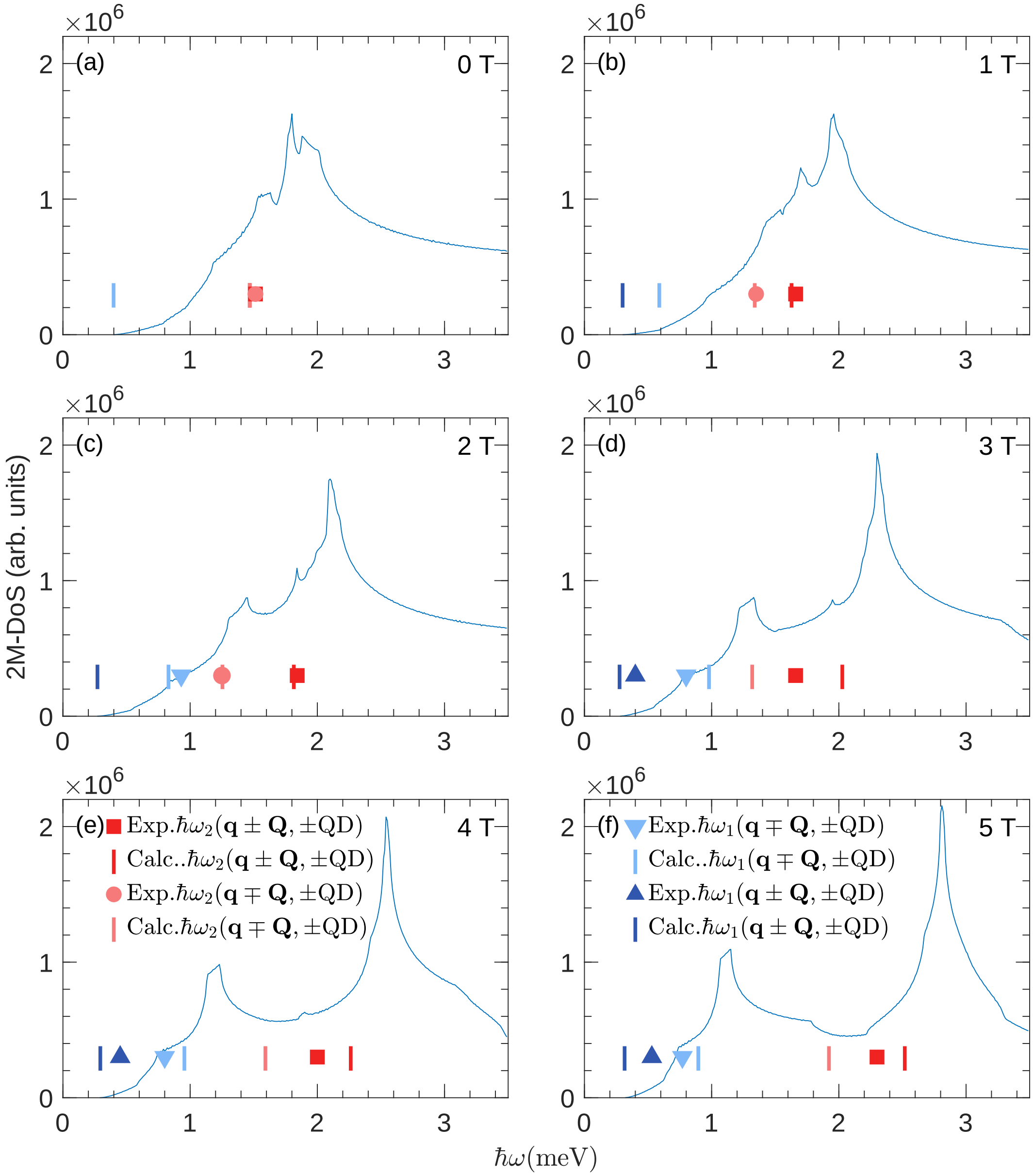

Fig. S6 One-dimensional cuts of the calculated 2-magnon density of state at Γ point. The calculated and observed one-magnon energies are indicated by the short vertical bars and the symbols.

refer to this simulation as the beam simulation.

A kernel containing the information about the sample shape, size, orientations along the axes, lattice constants, and UB matrix is then generated for the second part of the Monte Carlo simulation. MCViNE loads the beam simulation, and it evaluates the scattering with the sample rotating it for the same angular range used for the measurement. Finally, the scattered neutron trajectories are recorded on the detector array, reduced, and placed on a regular grid within the same momentum and energy transfer ranges used for the data. The resolution ellipsoids in the 4-dimensional $\mathbf{q}$-$\hbar\omega$ space are then simulated, fitted, and the resulting FWHMs saved in the final output file. The finer the grid, the longer these simulations will take. To achieve proper statistical average simulations have been performed with 10 million neutrons saved in the beam simulation.

The calculated one-magnon spectra, convoluted by the resolution ellipsoids, and integrated over the $\mathbf{q}$ range as indicated in Table S1 are shown in Figs. 2g-2l in the main article. Constant $\mathbf{q}$ cuts of the calculated spectra, convoluted by the resolution function, and integrated over the $\mathbf{q}$ range indicated in Table S1 at K, Γ, and M points, are shown by symbols in Figs. S7(a)-S7(c). The black solid curves represent the fitting results from the multiple Gaussians. Each individual Gaussian is represented by a different color; the areas represented in deep and light blue are $\hbar\omega_1(\mathbf{q}\pm\mathbf{Q}, \pm\text{QD})$ and $\hbar\omega_1(\mathbf{q}\mp\mathbf{Q}, \pm\text{QD})$ modes. Those shown in deep and light red are $\hbar\omega_2(\mathbf{q}\pm\mathbf{Q}, \pm\text{QD})$ and $\hbar\omega_2(\mathbf{q}\mp\mathbf{Q}, \pm\text{QD})$ modes. The fit to the data is reasonable, and it is safe to assume that the instrumental resolution function is approximately Gaussian. $\hbar\omega$ dependence of Full Width at Half Maximum (FWHM) of the Gaussian, denoted as FWHM_G, for each mode is indicated by the symbols in Figs. S7(d)-S7(f). FWHM_Gs at the energies of the observed excitations in Figs. 3**a**, 3**c**, and S1(a) are estimated from the interpolation of the data in Figs. S7(d)-S7(f). The measured constant $\mathbf{q}$ cuts are, thus, analyzed by Lorentzian convoluted by Gaussian with the width of FWHM_G.

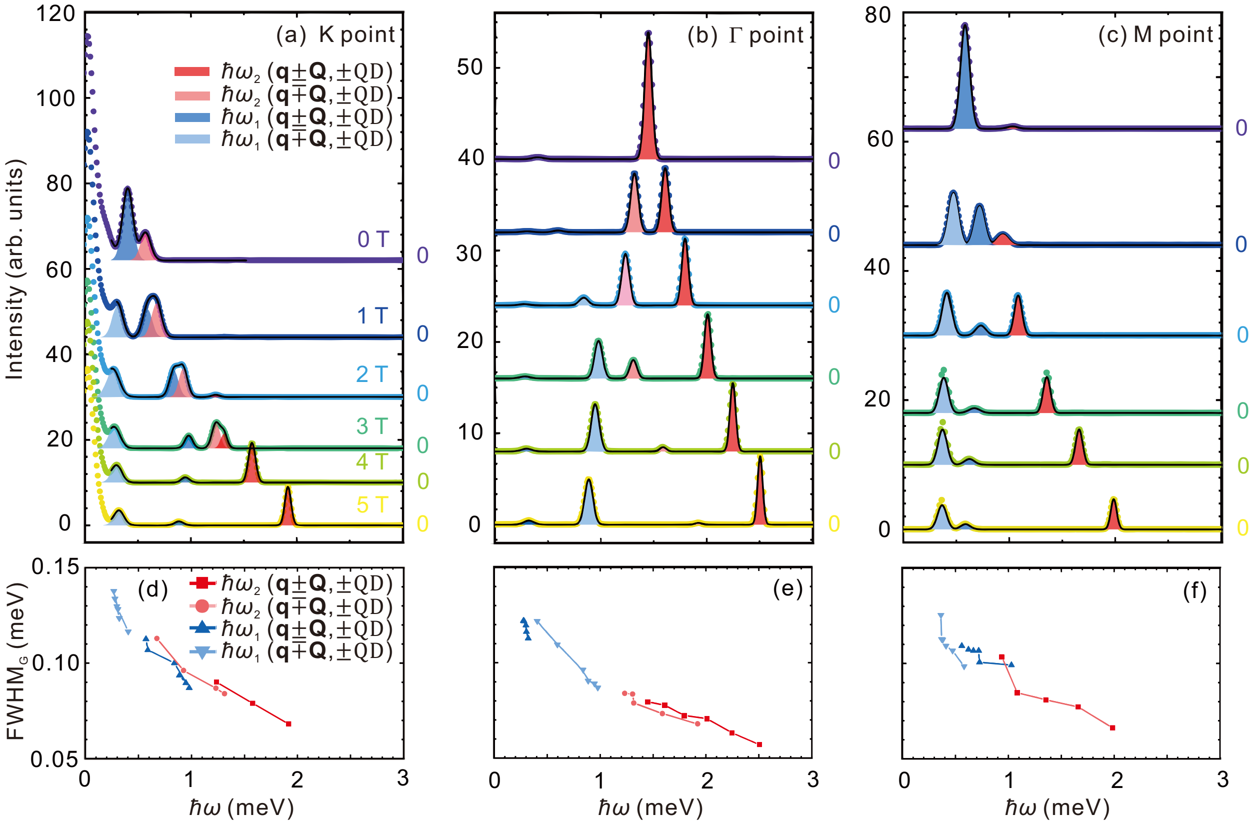

Fig. S7 (a)-(c) The symbols indicate constant **q** cuts of the calculated spectra convoluted by the resolution function at K point in (a), Γ point in (b), and M point in (c). The solid curves are the fitting results by multiple Gaussians. The colored area indicates the component of the Gaussians, the central energy of which is the eigenenergy of the mode as defined in the legend. (d)-(f) $\hbar\omega$ dependence of FWHM of the Gaussian, denoted as $\mathrm{FWHM_G}$ for each mode.

[1] N. Suzuki, J. Phys. Soc. Jpn. **52**, 1002 (1983).

[2] D. Petitgrand, B. Hennion, P. Radhakrishna, C. Esrcibe, and S. Legrand, Recent Developments in Condensed Matter Physics, edited by J. T. Devreese, L. F. Lemmens, V. E. Van Doren, and J. Van Royen **4**, 205 (1981).

[3] M. Matsumoto, S. Hayashida, and T. Masuda, J. Phys. Soc. Jpn. **89**, 034710 (2020).

[4] M. Matsumoto, J. Phys. Soc. Jpn. **83**, 084704 (2014).

[5] I. Affleck, Phys. Rev. Lett. **62**, 474 (1989).

[6] I. Affleck and G. F. Wellman, Phys. Rev. B **46**, 8934 (1992).

[7] M. L. Plumer and A. Caillé, Phys. Rev. Lett. **68**, 1042 (1992).

[8] L. Stoppel, S. Hayashida, Z. Yan, A. Podlesnyak, and A. Zheludev, Phys. Rev. B **104**, 094422 (2021).

[9] P. A. Maksimov, M. E. Zhitomirsky, and A. L. Chernyshev, Phys. Rev. B **94**, 140407 (2016).

[10] D. Macdougal, S. Williams, D. Prabhakaran, R. I. Bewley, D. J. Voneshen, and R. Coldea, Phys. Rev. B **102**, 064421 (2020).

[11] N. Wada, K. Ubukoshi, and K. Hirakawa, J. Phys. Soc. Jpn. **51**, 2833 (1982).

[12] N. Suzuki, J. Phys. Soc. Jpn. **59**, 2947 (1990).

[13] S. H. Do, H. Zhang, T. J. Williams, T. Hong, V. O. Garlea, J. A. Rodriguez-Rivera, T. H. Jang, S. W. Cheong, J. H. Park, C. D. Batista, and A. D. Christianson, Nat. Commun. **12**, 5331 (2021).

[14] J. Ma, Y. Kamiya, T. Hong, H. B. Cao, G. Ehlers, W. Tian, C. D. Batista, Z. L. Dun, H. D. Zhou, and M. Matsuda, Phys. Rev. Lett. **116**, 087201 (2016).

[15] K. Amaya, M. Ishizuka, T. Nakagawa, S. Saratani, T. Sakakibara, S. Takeyama, K. Nakao, T. Goto, N. Miura, Y. Unno, and Y. Ajiro, J. Phys. Soc. Jpn. **57**, 38 (1988).

[16] J. Y. Y. Lin, H. L. Smith, G. E. Granroth, D. L. Abernathy, M. D. Lumsden, B. Winn, A. A. Aczel, M. Aivazis, and B. Fultz, Nucl. Instrum. Meth. A **810**, 86 (2016).

[17] J. Y. Y. Lin, F. Islam, G. Sala, I. Lumsden, H. Smith, M. Doucet, M. B. Stone, D. L. Abernathy, G. Ehlers, J. F. Ankner, and G. E. Granroth, J. Phys. Commun. **3**, 085005 (2019).
